# Associations between Daily Ambient Air Pollution and Pulmonary Function, Asthma Symptom Occurrence, and Quick-Relief Inhaler Use among Asthma Patients

**DOI:** 10.3390/ijerph19084852

**Published:** 2022-04-16

**Authors:** Monika Ścibor, Bartosz Balcerzak, Andrzej Galbarczyk, Grazyna Jasienska

**Affiliations:** Department of Environmental Health, Faculty of Health Sciences, Jagiellonian University Medical College, 31066 Krakow, Poland; monika.scibor@uj.edu.pl (M.Ś.); bartosz.balcerzak@uj.edu.pl (B.B.); jasienska@post.harvard.edu (G.J.)

**Keywords:** PM_10_, PM_2.5_, air quality, respiratory disease, asthma, environmental health

## Abstract

Particulate matter (PM) is harmful to human health, especially for people with asthma. The goal of this study was to enhance the knowledge about the short-term effects of daily air concentrations of PM on health outcomes among asthma patients. The novelty of this study was the inclusion of a homogeneous group of patients (N = 300) with diagnosed and partly controlled asthma. Patients recorded their symptoms, asthma quick-relief inhaler use, and peak expiratory flow (PEF) measurements in a diary for two weeks. Data on particulate air pollution were obtained from stationary monitoring stations. We have shown that particulate pollutants (PM_10_ and PM_2.5_) are associated with significant deterioration of PEF and an increase in the frequency of early asthma symptoms, as well as asthma quick-relief inhaler use. These effects are observed not only on the day of exposure, but also on the following day. For public health practice, these results support the rationale for using peak-flow meters as necessary devices for proper asthma self-management and control, especially in locations where the air is polluted with particles. This may decrease the number of asthma patients seeking medical help.

## 1. Introduction

Particulate matter (PM) has a negative impact on human health [1,2]. For instance, exposure to PM causes respiratory diseases due to induction of oxidative stress and, consequently, increased inflammation [3]. As regards the general population, the increase in coarse (PM_10_) and fine (PM_2.5_) particles increases the number of doctor’s house calls for respiratory diseases [4] and the number of general practice consultations [5]. Short-term exposure to PM_2.5_ [6,7] or PM_10_ [8] is also associated with the increase in emergency department visits caused by asthma attacks. Both fractions of particles have an adverse effect on asthma hospitalization [9]. High PM_2.5_ and PM_10_ concentrations may also negatively affect the quality of life of people with asthma [10,11].

Studies of individuals with asthma exposed to particulate air pollutants should also include medication use, respiratory function, and the occurrence of symptoms. This research is essential not only for assessing the health status of vulnerable groups in the population, but it can also lead to improvement of prevention guidelines. The health status might be assessed by use of asthma medication, including quick-relief inhalers [12]. Quick-relief inhalers are used as needed to provide relief of symptoms of asthma exacerbations. Respiratory function might be easily assessed by monitoring peak expiratory flow (PEF). PEF measurements are very simple measures of the maximal flow rate that can be achieved during forceful expiration following full inspiration [13]. PEF is an objective, widely available measurement of pulmonary function and can be used at the patient’s home. PEF monitoring can also indicate the deterioration of respiratory function during poor quality of air earlier than asthma exacerbations/symptoms and consequently asthma quick-relief inhaler use [14].

Symptoms such as coughing and shortness of breath appear as the first signs of health problems; they are used along with measurements of respiratory function for self-monitoring, which, in addition to regular medical examination, is one of the most important components of asthma control by physicians [15]. Monitoring these symptoms is also crucial for the patient’s asthma self-management [16], the goal of which is to make a timely decision to use quick-relief inhalers. As demonstrated in studies, appropriate self-management benefits not only the individual, but the benefits are also apparent at the population level: it improves asthma control rates, significantly reduces the number of unplanned consultations and emergency department visits, and nearly halves the risk of hospitalization [17]. Thus, for both exploratory and practical reasons, it is very important to have a well-established knowledge of which symptoms appear in response to poor air quality, as a marker of declining health and as a signal to take relief medication.

Cross-sectional studies have shown that the concentration of PM in the air affects the prevalence of asthma symptoms [18], with this effect observed in adults and not in children [19]. The results of panel studies are less consistent. Some studies found a significant relationship between particulate pollution and the frequency of symptoms or lung function [20,21], while others found no such association [22,23].

Further, in some studies, the association between particulate pollution concentrations and respiratory symptoms or functions was not confirmed [23]. In other studies, the significant effect was limited only to coarser particulates less than 10 mm in diameter [21,24] or the significance of the effect was confirmed for symptoms, but not for respiratory function measurements such as the PEF [25].

The goal of the present study was to enhance existing knowledge about the short-term relationship between concentrations of particulate pollution (PM_10_ and PM_2.5_) in the air and the health outcomes important in asthma self-management and control. The aim was also to check how the air quality in the same day, previous day, and the previous two days affect asthma patients.

## 2. Materials and Methods

At first, 349 people were invited to take part in the study. All were patients suffering from bronchial asthma and had been treated in one of two separate allergy treatment clinics in Krakow, Poland. This recruitment process took place between June 2013 and May 2015. Krakow is the city where the air quality, particularly with respect to PM, was one of the worst in Europe at the time of our data collection [26,27].

Each recruited individual was an adult (>18 years old) residing in Krakow with partially controlled asthma. Partially controlled asthma is defined as a patient having experienced one or two of the following asthmatic features in the previous four-week period: daytime symptoms occurring more than twice weekly, quick-relief medication needed more than twice weekly, night waking, and activity limitation due to asthma [15]. All patients chosen for this study met the inclusion criteria. Each signed an informed consent form agreeing to participate in the survey and declaring that they would remain in the city during the 14-day observation period. Each study participant under observation was provided with the patient’s paper diary and a peak-flow meter. During the first visit, the physician specializing in allergic diseases conducted the interview and recorded the first part of the patient’s diary: patient ID, the starting date of observation, and other information regarding each patient’s date of birth, place of residence, education, smoking status, and what types of treatments for asthma the patient had used.

The second part of the patient’s diary was intended to be self-filled by the patient during the 14 days of observation. The study participants completed two tables daily by entering the results of peak expiratory flow (PEF) measurement in the morning and evening, then supplemented the rest of the diary by answering the following questions [28]:

- Whether the symptoms of asthma, such as cough or shortness of breath, wheezing, tight chest, had occurred during the day (which ones and under what circumstances).
- Whether at night the respiratory discomfort caused night-time waking due to asthma (nocturnal awakenings).
- Whether any symptoms had happened in the last 24 h: headaches, muscle pain, fever, runny nose (suggestive infections as a cause of worsening disease control).
- Whether any so-called emergency medicine had been used (asthma quick-relief inhaler): what kind? How many times?
- Whether they spend time outdoors, how many hours, street/district where most time was spent outdoors.

In the study, a mechanical mini-Wright peak-flow meter was used, which showed measurement accuracy in accordance with the international standardization organization ISO 23747:2015. The accuracy of PEF measurements can translate into the reliability of the results of clinical trials; therefore, particular importance was placed on training patients to perform these measurements. In addition, written instructions for performing PEF measurements were given to each study participant. PEF measurements were made according to the following standards: twice a day: morning and evening, each measurement was taken three times [29].

Using the data from each of the air-quality monitoring stations of the Voivodeship Inspectorate of Environmental Protection in Krakow, the daily concentrations of PM_2.5_ and PM_10_ were recorded for each patient during the 14 days of observation. The air-quality monitoring station situated nearest to each patient’s declared outdoor place of stay was assigned to each study participant during the study, in order to assess their exposure to PM.

We eventually used the data from 300 patients for analysis, ranging from 20 to 80 years in age (mean: 53 years, standard deviation (SD): 15.3 years): 145 women (48.3%) and 155 (51.7%), respectively. Of the original 349 patients, forty-nine had to be excluded from the study due to: leaving the city during the observation period; reporting symptoms of infection; failing to fill out or losing their observation journals. This study was performed under the auspices and approval of the Bioethics Committee of the Jagiellonian University (number KBET/167/B/2012). As we had PEF measurements as well as assessments of asthmatic symptoms, such as cough or shortness of breath, wheezing, tight chest, night-time waking due to asthma, or asthma quick-relief inhaler use for each patient from 14 consecutive days, generalized estimation equations (GEE) models were used to verify their association with airborne PM_10_ and PM_2.5_. GEE with normal distribution and identity link function when PEF measurement was considered as a dependent variable and GEE with binomial distribution and logit link function when the potential impact of airborne pollutants on the risk of asthma symptoms were assessed. An exchangeable correlation matrix was assumed, as we took into account that measurements within one patient will be correlated on the same level. PM_2.5_ and PM_10_ were used in separate models because they were highly correlated (r = 0.975, *p* < 0.001).

Our main interest was to verify the short-term impact of PM on the evening PEF level and occurrence of asthma symptoms. Therefore, the pollution levels from the same day (lag0), the previous day (lag1), and the previous two days (lag2) were considered. Finally, the model with the pollutant level at the same day and the change from the day before was used. All models were adjusted to age, gender, smoking, and heating season (defined as between 15th September–15th April during a year). All tests were two-tailed, and statistical significance was set at *p*-value < 0.05. All analyses were performed using PS IMAGO PRO 4.0 with IBM SPSS Statistics version 24 (Predictive Solutions Sp. z o.o. Krakow, Poland).

## 3. Results

All characteristics were presented as means with SD for continuous variables or frequency with percentage distribution for categorical variables, respectively (Table 1). Daily PM_10_ and PM_2.5_ exposure averaged 65 µg/m^3^ (median = 54 µg/m^3^, range: 9–256 µg/m^3^) and 45 µg/m^3^ (median = 37 µg/m^3^, range: 6–192 µg/m^3^), respectively. Concentrations were higher in the heating season compared to the non-heating one [PM_10_ median and interquartile range (IQR): 67 µg/m^3^ (43–102 µg/m^3^) vs. 28 µg/m^3^ (22–35 µg/m^3^); PM_2.5_ median and IQR: 46 µg/m^3^ (29–70 µg/m^3^) vs. 17 µg/m^3^ (13–23 µg/m^3^)]. All patients declared that they spent 2–3 h a day outdoors, on average.

The average PEF value was 364.2 ± 112.52 L/min, with a range from 120 to 800 L/min. Cough or shortness of breath was reported for 31.5% of measurements, wheezing for 6%, tight chest for 15%, night-time waking due to asthma for 18.5%, and asthma quick-relief inhaler use for 17.6% of measurements.

Both PM_10_ and PM_2.5_ levels significantly influenced the evening PEF parameter. Higher concentrations of PM_10_ or PM_2.5_ at the same day (lag0) as measurement were associated with lower PEF values (−0.90 L/min and −1.11 L/min by 5 µg/m^3^ increase in PM_10_ and PM_2.5_, respectively; *p* < 0.001). A similar association was observed with particulate concentrations from the day before (lag1). However, the effect was lower (−0.74 L/min and −0.92 L/min by 5 µg/m^3^ increase in PM_10_ and PM_2.5_, respectively; *p* < 0.001). The PM_10_ level, but not PM_2.5_, recorded 2 days before PEF measurement (lag2), was associated with PEF values. In the models with the pollutant level at the same day (lag0) and the change from the day before (lag0–lag1), we observed that higher PM_10_ and PM_2.5_ concentrations at the same day and an increase in particulate concentration from the day before negatively influenced the PEF parameter (Table 2).

The effect of particulate concentrations during the observation day (lag0) was also observed for the risk of asthma symptoms such as cough or shortness of breath and tight chest, as well as asthma quick-relief inhaler use (Table 3). The risk of cough or shortness of breath was 4% and became 5% higher for each 5 µg/m^3^ increase in PM_10_ or PM_2.5_, respectively (*p* < 0.001). The risk of tight chest was 4% higher by each 5 µg/m^3^ increase in PM_10_ or PM_2.5_ (*p* < 0.001). The risk of asthma quick-relief inhaler use was 5% and 6% higher by each 5 µg/m^3^ increase in PM_10_ or PM_2.5_, respectively (*p* < 0.001). A similar, but weaker effect was observed with the concentration of PM_10_ or PM_2.5_ from the day before the observation day (lag1).

The risk of the occurrence of wheezing was not related to PM_10_ or PM_2.5_ concentrations. The risk of nocturnal awakenings was 3% higher for each 5 µg/m^3^ increase in PM_10_ or PM_2.5_ during the day before (*p* < 0.001) but was not affected by the PM_10_ or PM_2.5_ concentrations recorded two days prior to observation. The risk of any of the asthma symptoms and medication use was not related to the air pollution concentrations recorded 2 days before observation (lag2) nor to the difference in air pollution concentrations between the day of observation and the day before (lag0–lag1).

## 4. Discussion

In undertaking this study, we intended to enhance the existing knowledge about the relationship between daily concentrations of particulate pollution (PM_10_ and PM_2.5_) in the air and short-term health outcomes. Such knowledge is important in asthma self-management and control. The novelty of this study was the inclusion of patients with controlled asthma, who were under permanent control of a medical doctor and were regularly taking prescribed medications. Previous studies investigated much more heterogenous groups that included people with significant differences in disease control. Our more rigorous methodological approach reduced potentially confounding effects of patients’ current health status; thus, it is not surprising that our results differ from some results of previous panel studies [23,30].

The results of our study are fairly consistent. They confirm that particulate pollutants, both PM_10_ and PM_2.5_, significantly affect the deterioration of respiratory function (PEF), the occurrence of asthma symptoms, and the use of medication. These effects are observed not only during the day of exposure, but also during the following day. The only symptom for which such an association was not confirmed is wheezing, although some studies reported such an association for PM_10_ [20,21]. Wheezing is the result of the airway narrowing; as such, it occurs later than other symptoms, and usually as a result of poor asthma management. When a patient exhibiting other symptoms uses a quick-relief inhaler, it reduces the likelihood of wheezing. Therefore, medication use may mask some severe symptoms of air-pollution exposure, such as wheezing [23].

It should be noted that apart from particulate pollution (PM_10_ and PM_2.5_), the concentration of gaseous air pollutants may be associated with the frequency of rescue inhaler use and the occurrence of asthma symptoms. The existence of such relationship has been confirmed for nitrogen dioxide (NO_2_) [20,31], ozone (O_3_) [32], or sulfur dioxide (SO_2_) [33]. One of the reasons for the difference between our results and those of previous panel studies could be the way exposure was estimated [20,21,23,30]. Although none of the cited studies used personal air pollution monitors, but rather, as in our study, data from stationary monitors, the number of stations in the city and their location in relation to the place of residence of the subjects may have had some impact. Some authors [23] have even expressed doubts that using data from stationary stations for exposure estimation purposes may lead to misclassification, resulting in a lack of significance of the relationship between particulate pollution and health effects. We used data from three stations, and in an earlier publication, we demonstrated that the data from those stations adequately reflected the subjects’ exposure to particulate pollution [26].

However, it should be emphasized that our exposure assessment method used for estimating PMs’ concentrations was in the places of residence of the study subjects, and therefore did not take into account their mobility. Recent studies suggest that exposure estimation models using data obtained from location-based services (such as global positioning system (GPS) trajectory data sets or social network logins) and PMs’ concentration maps derived from satellite-station-hybrid models may better assess study participants’ exposure [34]. However, some obstacles to such a study design are to be expected, including a lack of consent from potential study participants to have their cell phones tracked.

The method used to measure the respiratory function can influence the results as well. Lung-function tests can be performed using either a spirometer or a peak-flow meter, and using both of these devices at the same time and on the same sample of subjects may provide different results [22]. The spirometer is routinely used in the diagnosis and assessment of asthma. Although this device is considered to be more accurate than a peak-flow meter and less prone to measurement error [22], its use in asthma monitoring by a patient is quite limited. A spirometer is a larger, more expensive, and more sophisticated device than a peak-flow meter, but it cannot be used by patients themselves and must be operated by a healthcare professional. The measurement of respiratory function with use of a spirometer is thus restricted to specific hours of operation of medical or research facilities. Limited access to spirometers means that it is not possible to measure many people at the same time (e.g., in the evening hours). In addition, the measurement does not take place at the subject’s place of residence and is usually preceded by traveling or walking which may affect the exposure itself. The use of a peak-flow meter does not come with these limitations. The risk of measurement error can be reduced by detailed patient education [22], as was done in our study.

Our results primarily support the rationale for using peak-flow meters as necessary devices for proper asthma self-management and control, especially in locations where the air is polluted with particles. Thus, physicians should recommend the use of these devices to their patients and train them in their proper use [22]. Deterioration of respiratory function and worsening of symptoms may be the signal to the patient that air quality is worsening, such that protective behaviors for reducing exposure to pollutants (e.g., limiting outdoor activities) should be implemented. It is well established that avoiding triggers is vital for adequate asthma control [35].

In addition, checking current air-quality information and forecasts of particulate matter air pollution to avoid triggers and reduce exposure can be considered as a coping behavior and an important part of asthma self-management. However, this behavior needs the support of a local pollution-information system, which is not only an important extension of air-quality monitoring nets, but also one of the most important reasons for their establishment. Such systems can use various tools, such as cell-phone applications, information boards placed in public spaces or on public transport, etc. While there are some costs associated with the development and maintenance of such systems, they allow for substantial cost savings. It has been estimated that cases of uncontrolled asthma cause a significant financial burden, which results in not only the costs of their treatment and the use of healthcare services, but also indirect costs, such as absence from work and temporary suspension of important social roles [36]. Air-quality information systems are not only dedicated to people with asthma but are also used by other community members to protect and sustain their own health.

## 5. Conclusions

Particulate pollutants (PM_10_ and PM_2.5_) may be associated with significant deterioration of PEF and increase the frequency of early asthma symptoms, as well as the use of asthma quick-relief inhalers. These effects can be seen not just on the day of exposure, but also the day after. These findings support the use of peak-flow meters as necessary devices for proper asthma self-management and control, especially in locations where the air is polluted. Moreover, access to current air-quality information and forecasting of air quality is needed in order to take preventive action during episodes of air pollution.

## Figures and Tables

**Table 1:** Characteristics of the study cohort.

Variable	N = 300
Gender [*n* (%)]	
Women	145 (48.3)
Men	155 (51.7)
Age [years] (Mean, SD)	53 (15.3)
Education [*n* (%)]	
Secondary or lower	173 (57.7)
Higher	127 (42.3)
Active smoker [*n* (%)]	31 (10.3)
Heating season [*n* (%)]	230 (76.7)

**Table 2:** Effect of PM_10_ and PM_2.5_ levels on the evening peak expiratory flow (PEF).

		PM_10_ (5 µg/m^3^) *		PM_2.5_ (5 µg/m^3^) *	
		B (95% CI)	*p*	B (95% CI)	*p*
**Model 1**	lag0	**−0.90 (−1.17; −0.63)**	**<0.001**	**−1.11 (−1.50; −0.73)**	**<0.001**
**Model 2**	lag1	**−0.74 (−0.98; −0.50)**	**<0.001**	**−0.92 (−1.25; −0.60)**	**<0.001**
**Model 3**	lag2	**−0.25 (−0.49; −0.003)**	**0.047**	−0.34 (−0.67; 0.004)	0.052
**Model 4**	lag0	**−1.07 (−1.37; −0.77)**	**<0.001**	**−1.33 (−1.75; −0.91)**	**<0.001**
	lag0–lag1	**−0.36 (−0.62; −0.10)**	**0.006**	**−0.46 (−0.84; −0.08)**	**0.017**

* Adjusted to age, gender, smoking, and heating season (Yes vs. No). B—beta coefficient; CI—confidence interval. Results in bold are statistically significant (*p* < 0.05).

**Table 3:** Effect of PM_10_ and PM_2.5_ levels on asthma symptoms and medication use.

		PM_10_ (5 µg/m^3^) *		PM_2.5_ (5 µg/m^3^) *	
		OR (95% CI)	*p*	OR (95% CI)	*p*
**Cough or shortness of breath**					
**Model 1**	lag0	**1.04 (1.03; 1.05)**	**<0.001**	**1.05 (1.03; 1.07)**	**<0.001**
**Model 2**	lag1	**1.02 (1.01; 1.03)**	**0.001**	**1.02 (1.01; 1.04)**	**0.001**
**Model 3**	lag2	1.00 (0.99; 1.01)	0.891	1.00 (0.99; 1.02)	0.757
**Model 4**	lag0	**1.04 (1.02; 1.05)**	**<0.001**	**1.05 (1.03; 1.07)**	**<0.001**
	lag0–lag1	1.00 (0.98; 1.01)	0.385	0.99 (0.98; 1.01)	0.440
**Wheezing**					
**Model 1**	lag0	1.01 (1; 1.03)	0.163	1.01 (0.99; 1.04)	0.178
**Model 2**	lag1	1.01 (0.99; 1.03)	0.268	1.01 (0.99; 1.04)	0.277
**Model 3**	lag2	1.00 (0.98; 1.01)	0.739	1.00 (0.98; 1.02)	0.960
**Model 4**	lag0	1.01 (1; 1.03)	0.142	1.02 (0.99; 1.04)	0.147
	lag0–lag1	1.01 (0.98; 1.03)	0.619	1.01 (0.98; 1.04)	0.610
**Tight chest**					
**Model 1**	lag0	**1.04 (1.02; 1.05)**	**<0.001**	**1.04 (1.03; 1.06)**	**<0.001**
**Model 2**	lag1	**1.02 (1.002; 1.03)**	**0.023**	**1.04 (1.02; 1.06)**	**<0.001**
**Model 3**	lag2	1.00 (0.98; 1.01)	0.421	0.99 (0.97; 1.01)	0.219
**Model 4**	lag0	**1.03 (1.02; 1.05)**	**<0.001**	**1.04 (1.02; 1.06)**	**<0.001**
	lag0–lag1	0.99 (0.98; 1.01)	0.196	1.00 (0.97; 1.01)	0.219
**Night-time waking due to asthma**					
**Model 2**	lag1	**1.03 (1.01; 1.04)**	**<0.001**	**1.03 (1.01; 1.05)**	**<0.001**
**Model 3**	lag2	1.00 (0.99; 1.02)	0.651	1.01 (0.99; 1.03)	0.326
**Asthma quick-relief inhaler use**					
**Model 1**	lag0	**1.05 (1.03; 1.06)**	**<0.001**	**1.06 (1.04; 1.08)**	**<0.001**
**Model 2**	lag1	**1.02 (1.01; 1.04)**	**<0.001**	**1.03 (1.01; 1.05)**	**0.001**
**Model 3**	lag2	1.00 (0.98; 1.01)	0.442	1.00 (0.98; 1.02)	0.813
**Model 4**	lag0	**1.04 (1.03; 1.06)**	**<0.001**	**1.06 (1.04; 1.08)**	**<0.001**
	lag0–lag1	0.99 (0.98; 1.01)	0.343	0.99 (0.97; 1.01)	0.419

* Adjusted to age, gender, smoking and heating season (Yes vs. No). CI—confidence interval. Results in bold are statistically significant (*p* < 0.05).

## Data Availability

The data presented in this study are available on request from the corresponding author. The data are not publicly available due to privacy restrictions.
